# Thirty Years of Kawasaki Disease: A Single-Center Study at the University Hospital of Lausanne

**DOI:** 10.3389/fped.2019.00011

**Published:** 2019-01-30

**Authors:** Marion de La Harpe, Stefano di Bernardo, Michaël Hofer, Nicole Sekarski

**Affiliations:** ^1^Pediatric Cardiology Unit, University of Lausanne, Lausanne, Switzerland; ^2^Pediatric Cardiology Unit, University Hospital of Lausanne (CHUV), Lausanne, Switzerland; ^3^Pediatric Rheumatology Unit, University Hospital of Lausanne (CHUV), Lausanne, Switzerland

**Keywords:** Kawasaki disease, coronary aneurysm, complications, cardiac sequelae, follow-up, prognosis

## Abstract

Kawasaki disease is an acute vasculitis with a particular involvement of the coronary arteries. Coronary artery aneurysms develop in 20% of untreated children. It has been shown that early treatment with intravenous immunoglobulins and aspirin decreases this risk to 5%, but the medium to long term prognosis of children with Kawasaki disease is still unclear. To determine the outcome of the disease and risk factors for poor evolution, we reviewed retrospectively the medical records of all patients with a diagnosis of Kawasaki disease at our Institution between 1981 and 2014. Among the 207 patients included in the study, 96 patients had coronary diameter anomalies (46.4%) at diagnosis and children with atypical ages for Kawasaki disease (<1 year or >10 year of age) were more often affected with aneurysms or dilatations. Eighty-four of them had complete regression of coronary aneurysms during the follow-up (87.5%) Absence of immunoglobulins in the acute phase was associated with less regression rate (57.1 vs. 92.2%), and boys had greater *z*-scores at last echocardiography, statistically significant for the left anterior descending artery. We found rare complications after the acute phase documented in our patient charts (only 3.8%). Recurrence of the disease occurred in 5 children (2.4%) and myocardial ischemia in 3 patients (1.4%), all with initial coronary aneurysm.

**Conclusion:** Medium to long term prognosis after Kawasaki disease is excellent. Boys, patients not treated with immunoglobulins or outside the usual age range are more at risk for an unfavorable outcome.

## Introduction

Kawasaki disease (KD) is an acute systemic vasculitis of early childhood considered the leading cause of acquired heart disease in children in developed countries. Cardiac lesions are a hallmark of KD and coronary artery aneurysms (CAA) develop in 20% of untreated children and can lead to coronary stenosis, myocardial infarction (MI), or sudden death. Pericarditis complicated by cardiac tamponade or myocarditis associated with myocardial dysfunction can also occur during the acute phase (1, 2).

Despite almost 50 years of research, the etiology of KD remains unknown. An infectious trigger which causes an excessive inflammatory response in genetically predisposed children is a widespread hypothesis, but no specific pathogen has been identified yet (3).

KD has been reported in almost every country, with variable incidence rates (4). Over the past decades, the worldwide incidence has been increasing in most countries. KD occurs predominantly in children <5 years of age, with a peak incidence between 12 and 24 months (5–10).

Without a specific diagnostic test, the diagnosis of KD is based on clinical criteria, established by the Japanese Ministry of Health Research Committee and adopted by the American Heart Association, which include fever >39°C for at least 5 days (or less if shortened by treatment for KD) plus four of the five main clinical features: rash, conjunctivitis, oral mucosal changes, changes in the extremities, lymphadenopathy. In some cases, KD presentation is incomplete and echocardiographic and laboratory findings have an important role in the diagnosis (11–14).

Conventional therapy associates intravenous immunoglobulin (IVIG) and high-dose aspirin (ASA). Early administration of IVIG reduces the risk of CAA to 5% (15–18). About 15–20% of patients are unresponsive to this initial treatment and will need repeat or additional treatment such as corticosteroids or infliximab (19, 20).

Mortality rates are low and the majority of patients are considered to have an excellent prognosis but long-term cardiovascular outcome is not totally defined (21–25). Only males with cardiac sequelae are known to have a higher mortality rate than the general population (26). However, even for patients with regressed CAA, there may be a possible cardiovascular dysfunction years after the acute phase, secondary to increased arterial stiffness, abnormal endothelial function, intimal thickening or calcification of coronary arteries (1, 11, 27–31). The clinical relevance of those changes is unclear and long-term follow up of patients depends on the degree of coronary involvement.

Because the prognosis of patients with a diagnosis of KD is not well defined, we decided to conduct this study to determine the characteristics and outcome of all patients at our Institution between 1981 and 2014 and to highlight risk factors for unfavorable evolution.

## Materials and Methods

### Patients and Study Design

This is a retrospective study including all patients with a diagnosis of pediatric KD, followed in the Pediatric Cardiology Unit at the University Hospital of Lausanne.

We analyzed retrospectively the medical records of all patients up to 18 years of age at diagnosis, with complete or incomplete KD and followed in the Pediatric and Adult Cardiology Unit of the University Hospital of Lausanne from January 1981 to March 2014. The following variables were documented: epidemiologic and clinical features, laboratory and echocardiographic data, treatment regimen, complications during the acute phase (shock, heart failure, cerebral injury, aseptic meningitis, uveitis, renal dysfunction, haemolysis, and pancreatitis) and during the follow-up (death, recurrence, ischemia, surgical or catheter coronary interventions).

Patients were excluded from the study if the diagnosis of KD was rejected or if there were missing information in their medical records.

The study protocol was approved by the Cantonal Ethics Committee Vaud.

### Definitions

A KD case was defined as: fever for 5 days or more (unless IVIG treatment was begun before the 5th day of fever), exclusion of any other diagnosis, and any four of the five principal clinical features. Incomplete KD was defined according to the guidelines of the American Heart Association as fever for 5 days or more, <4 diagnosis criteria and echocardiographic abnormalities or a set of suspect laboratory criteria (1).

First day of fever was defined as day 1 of illness.

### Cardiovascular Assessment

Cardiovascular complications, including coronary artery lesions, pericarditis, myocarditis, valvular regurgitations and heart failure, were assessed during the acute phase and during the follow-up at 3 different periods of time (6 month to 18 months, 3 to 6 years, 8 years and more). The coronary involvement (dilatation, small, medium, giant CAA) was determined by the z-scores adjusted to patients' body surface area (BSA) (Boston formula), if available. We used the z-score stratification proposed by AHA guidelines (2017) to determine the degree of coronary involvement (32):

1. No involvement: Z score always <2
2. Dilation only: Z score 2 to <2.5; or if initially <2, a decrease in Z score during follow-up ≥1
3. Small aneurysm: Z score ≥2.5 to <5
4. Medium aneurysm: Z score ≥5 to <10, and absolute dimension <8 mm
5. Large or giant aneurysm: Z score≥10, or absolute dimension ≥8 m

If z-scores could not be calculated because of missing details about coronary diameters, the degree of involvement was based on cardiologist report.

### Statistical Analysis

Statistical analysis was performed using the statistical software STATA 14. Continuous variables are reported as median and range (minimum, maximum). Categorical data are reported by percentage. Pearson's chi-squared test was used to compare distribution between groups, separated by sex, age, treatment regimen, KD presentation (complete vs. incomplete). A *p* < 0.05 was considered statistically significant.

## Results

### Patients' Characteristics

During the study period, 229 patients with KD were diagnosed at the University Hospital of Lausanne. Twenty-two were excluded because of missing information in their medical file.

Of the 207 children included, 58.9% were male (*n* = 122), with a sex ratio of 1.43.

**Figure 1:**
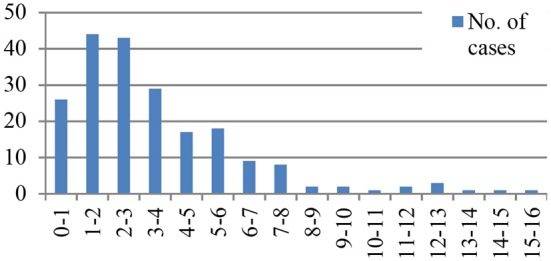
Age at onset of KD.

The median age at onset of KD was 32 months (range 1 month to 15 years). The majority of patients were less than 5 years of age (76.8%, *n* = 159). Age distribution is shown in Figure 1.

KD was complete in 146 cases (70.5%) and incomplete in 61 cases (29.5%). The annual number of incomplete KD increased after 1996: only 7 (17%) children were diagnosed with incomplete KD from 1981 to 1996 compared to 54 (32.5%) cases after 1996. Patients with incomplete KD were younger at diagnosis than patients with complete KD, but with no statistically significant difference (26 vs. 35 months, *p* = 0.08). The diagnosis, and therefore treatment, of patient with incomplete KD was delayed compared to complete KD (median duration before diagnosis 6.5 vs. 8 days, *p* = 0.007).

The diagnostic criteria and other clinical features are summarized in Table 1. There was no statistically significant difference in disease presentation according to sex or age.

**Table 1:** Clinical features at presentation.

**Diagnostic criteria**		**Other clinical features**	
Polymorphus rash	*n* = 176 (85%)	GI tract (diarrhea, vomiting, abdominal pain, hematemesis)	*n* = 121 (58.4%)
Changes in lips and oral cavity	*n* = 161 (77.8%)	Respiratory/ORL (cough, dyspnea, pneumonia, sinusitis, epistaxis, pharyngitis)	*n* = 60 (30.4%)
Conjonctival injections	*n* = 159 (76.8%)	Musculoskeletal (arthralgia, myalgia, joint effusion)	*n* = 30 (14.6%)
Changes of the extremities	*n* = 144 (69.9%)	Neurological (seizure, meningism, headache, dizziness, lethargy, photophobia)	*n* = 24 (11.6%)
Cervical adenopathy	*n* = 136 (65.7%)	Genito-urinary (hematuria, proteinuria, oliguria, urinary infection)	*n* = 18 (8.7%)
		Cardiac (murmur)	*n* = 2 (1%)

### KD Occurrence

The University Hospital of Lausanne is a leading tertiary center that covers an overall population of around 1,400,000 person. Every child within this area with a suspected diagnosis of KD is referred for echocardiographic evaluation to its pediatric cardiology unit.

**Figure 2:**
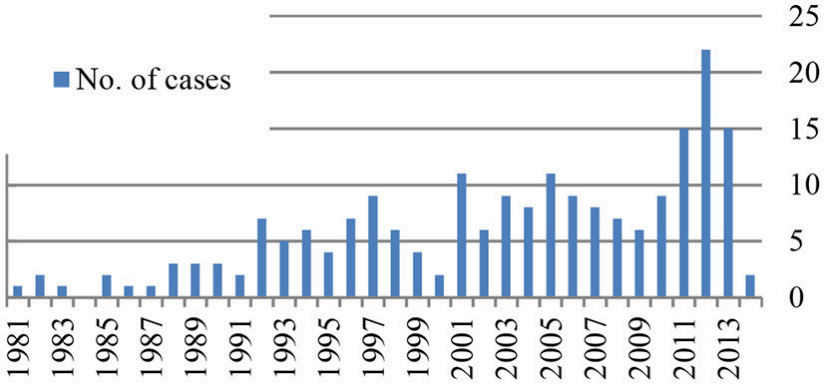
Occurrence rates.

As shown in Figure 2, KD was diagnosed at least in one child every year except in 1984, and there is an increase in the number of patients identified over the years with a peak of 22 patients in 2012.

A non-significant seasonal variability was noted, with a highest incidence in winter (29.5% of KD occurred in winter, which represents 61 patients).

### Treatment

**Figure 3:**
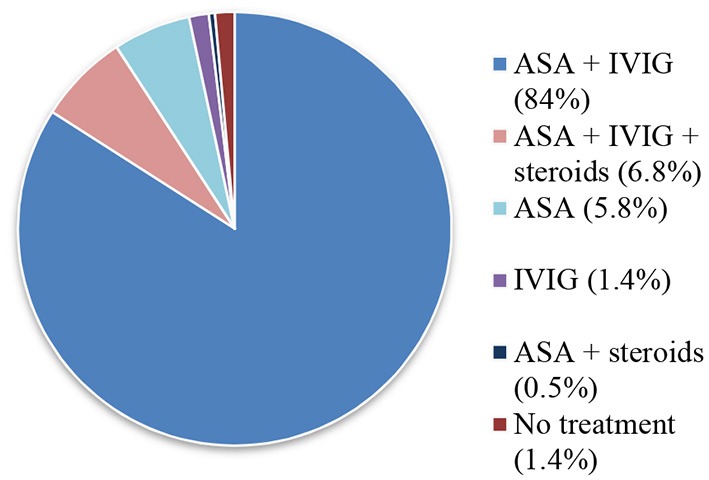
Treatment during the acute phase.

Treatment during the acute phase of KD is summarized in Figure 3. All patients received a first infusion of IVIG, except 16 children (7.7%). Half of them were diagnosed before IVIG was available at our Institution. The other half was diagnosed after more than 7 days of evolution.

One hundred and Sixty Five patients (86.4%) received a single IVIG infusion of 2 g/kg, the other patients received 0.4 g/kg for 4–5 days (13.1%, *n* = 25) or 1 g/kg for 2 days (0.5%, *n* = 1). IVIG therapy was well tolerated and only 8 patients (3.9%) developed side effects (allergic reaction, hypotension).

A majority of children also received ASA (97.1%, *n* = 201). High dose of ASA (80–100 mg/kg/day) was given in the majority of cases (86.1%, *n* = 173). This dose was usually reduced to 5 mg/kg/day after the acute phase.

Fifteen treated patients (7.2%) also received steroids during their hospitalization. Reasons for using steroids were either severe coronary anomalies or associated severe complications such as shock, respiratory distress, pancreatitis, renal insufficiency, ascites, pleural effusion, and pancytopenia.

Antibiotics were given in 31 cases (15% of treated patients), concomitantly or before diagnosis.

Three patients (1.4%) were diagnosed with KD after more than 2 weeks of evolution and were not treated either with IVIG or ASA since they had no residual inflammatory changes.

The majority of patients (58.5%, *n* = 121) were treated before day 7 of illness (median: day 7; range: day 1–39). However, 22.7% of patients (*n* = 47) did not received a treatment before the 10th day of illness.

Thirty-four children (16.4%) needed a 2nd therapy because of persistent or recrudescent fever after the initial treatment: 24 patient a 2nd IVIG infusion, 8 patients received steroids associated to IVIG. Infliximab was added to this latter treatment once. Steroids alone were used in 1 case (3%).

Three children needed a 3rd treatment for persistent fever (1.4%): a 3rd IVIG infusion (*n* = 1), corticosteroid associated to Anakinra (*n* = 1) and Infliximab (*n* = 1).

There was no difference in gender, age distribution or KD presentation between IVIG responders and non-responders.

Information about treatment after the acute phase was available for 200 children (96.6%). Patients were treated with ASA more than 1 year after the onset of KD in 29 cases (14%). The median duration of treatment after the acute phase was 2 months (range 0 month to 34 years). At the end of the study period, 11 patients (5.3%) were still under treatment. Every patient received low-dose (5–10 mg/kg/day) ASA and additional acenocoumarol was given in 1 child with persistent giant CAA.

### Echocardiography

Results of echocardiography during the acute phase of KD were available for all patients except for 3 Swiss children who were diagnosed with KD and treated abroad during their holidays. The echocardiography findings at diagnosis are summarized in Table 2.

**Table 2:** Echocardiography results at diagnosis.

**Perivascular brightness of coronary arteries**	*n* = 109 (52.6%)
**Coronary dilatation**	*n* = 16 (7.7%)
**Coronary aneurysm:**	
- Small	*n* = 41 (19.8%)
- Medium	*n* = 32 (15.4%)
- Giant	*n* = 7 (3.4%)
**Pericarditis:**	
- Pericardial effusion	*n* = 54 (26.1%)
- Cardiac tamponade	*n* = 2 (1%)
**Valvular disease**	*n* = 41 (19.8%)
**Myocarditis:**	
- Decreased ejection fraction of the left ventricle	*n* = 12 (5.8%)
- Congestive heart failure	*n* = 3 (1.4%)
**Arrhythmia**	*n* = 4 (1.9%)

Z-scores could be calculated in 171 echocardiograms (82.6%), the other 36 did not report the exact diameters of the coronary arteries. After the z-score calculation, the degree of coronary involvement was modified from the echocardiographic report in 90 cases (52.6% of the patients with z-score). Cardiologists underestimated the coronary artery lesions in 70 patients (40.9%) and overestimated them in 20 patients (11.7%).

The initial echocardiography showed anomalies in 136 cases (65.7%). Perivascular brightness of coronary arteries was the most common finding (52.6%, *n* = 109), followed by changes in coronary arteries diameter (46.4%, *n* = 96).

Isolated left coronary artery lesions (25.6%, *n* = 53) were more frequent than isolated right coronary artery lesions (4.8%, *n* = 10). Bilateral abnormalities occurred in 33 patients (15.9%).

In order of decreasing frequency, the sites of left coronary involvement were the left anterior descending coronary artery (LAD) (51.1% of left coronary artery lesions, *n* = 44), both LAD and left main coronary artery (LMCA) (25.6%, *n* = 22), isolated LMCA (23.2%, *n* = 20). LMCA did not present any giant CAA. Multiple CAA on the same coronary artery occurred in only one patient (0.5%).

No patient suffered from MI during the acute period.

Patients with atypical ages for KD (<1 or >10 year of age) had a higher incidence of changes in coronary arteries diameter (atypical age: 62.8% of coronary artery lesion vs. age 1–10 year: 43%, *p* = 0.01, see Table 3). Giant CAA occurred in 11.4% of patients with an atypical age and 1.7% in the 1–10 year old group.

**Table 3:** Coronary involvement according to age, sex and IVIG treatment.

		**Study population (*n* = 207)**	**Age 1-10 year (*n* = 172)**	**Atypical age (*n* = 35)**	***P-*value**	**Males (*n* = 122)**	**Females (*n* = 85)**	***P-*value**	**IVIG (*n* = 191)**	**No IVIG (*n* = 16)**	
CAA at diagnosis	Normal	111 (53.6%)	98 (56.9%)	13 (37.1%)	**0.01**	61 (50%)	50 (58.8%)	NS	100 (52.3%)	11 (68.7%)	NS
	Dilatation	16 (7.7%)	14 (8.1%)	2 (5.7%)	**0.01**	8 (6.5%)	8 (9.4%)	NS	15 (7.8%)	1 (6.2%)	NS
	Small CAA	41 (19.8%)	34 (19.7%)	7 (20%)	**0.01**	26 (21.3%)	15 (17.6%)	NS	39 (20.4%)	2 (12.5%)	NS
	Medium CAA	32 (15.4%)	23 (13.4%)	9 (25,7%)	**0.01**	22 (18%)	10 (11.7%)	NS	32 (16.7%)	0 (0%)	NS
	Giant CAA	7 (3.4%)	3 (1.7%)	4 (11.4%)	**0.01**	5 (4.1%)	2 (2.3%)	NS	5 (2.6%)	2 (12.5%)	NS
CAA at last follow-up	Normal	194 (93.7%)	162 (94.2%)	32 (91%)	NS	111 (90.9%)	83 (97.6%)	NS	181 (94.7%)	13 (81.2%)	NS
	Dilatation	1 (0.5%)	1 (0.58%)	0 (0%)	NS	0 (0%)	1 (1.2%)	NS	1 (0.5%)	0 (0%)	**<0.001**
	Small CAA	5 (2.4%)	4 (2.3%)	1 (2.9%)	NS	5 (4.1%)	0 (0%)	NS	5 (2.6%)	0 (0%)	**<0.001**
	Medium CAA	3 (1.4%)	3 (1.74%)	0 (0%)	NS	3 (2.4%)	0 (0%)	NS	3 (1.6%)	0 (0%)	**<0.001**
	Giant CAA	3 (1.4%)	2 (1.16%)	1 (2.9%)	NS	2 (1.6%)	1 (1.2%)	NS	1 (0.5%)	2 (12.5%)	**<0.001**
	Tortuous	1 (0.5%)	0 (0%)	1 (2.9%)	NS	1 (0.8%)	0 (0%)	NS	0 (0%)	1 (6.2%)	**<0.001**

*Bold values are values that are statistically significant*.

Valvular involvement occurred in 19.8% of patients (*n* = 41) and most often affected the mitral valve (87.8% of valvular dysfunction, *n* = 36). Fifty patients (27%) suffered from pericarditis.

### Death

Of the 207 patients, 1 died during the acute phase. This corresponds to a mortality rate of 0.5%. The deceased patient was a 33 months old boy. He developed cardiac tamponade, pulmonary hypertension and vena cava thrombus treated with thrombolysis and heparin. He died of subsequent cerebral hemorrhage 13 days after onset of KD.

### Follow-Up

Echocardiography report after the acute phase of KD was available in the majority of cases (97.1%, *n* = 201). The patients were followed up for a median duration of 21 months (range 0 month to 30 years). The patients without echocardiography follow-up were 6 children diagnosed between 1982 and 2010 who did not have any CAA and no subsequent follow-up appointment was planned for them.

Three different time frames from the time of diagnosis were selected for the echocardiographic follow-up: 6 to 18 months, 3 to 6 years, 8 years and more. A follow-up echocardiogram at 6 to 18 months was performed in 137 patients (66.2%, median time 12 months). Forty-five patients had an echocardiography follow-up at the second time frame (21.7%, median time 4 years) and 34 patients after 8 years or more (16.4%, median time 10 years).

**Figure 4:**
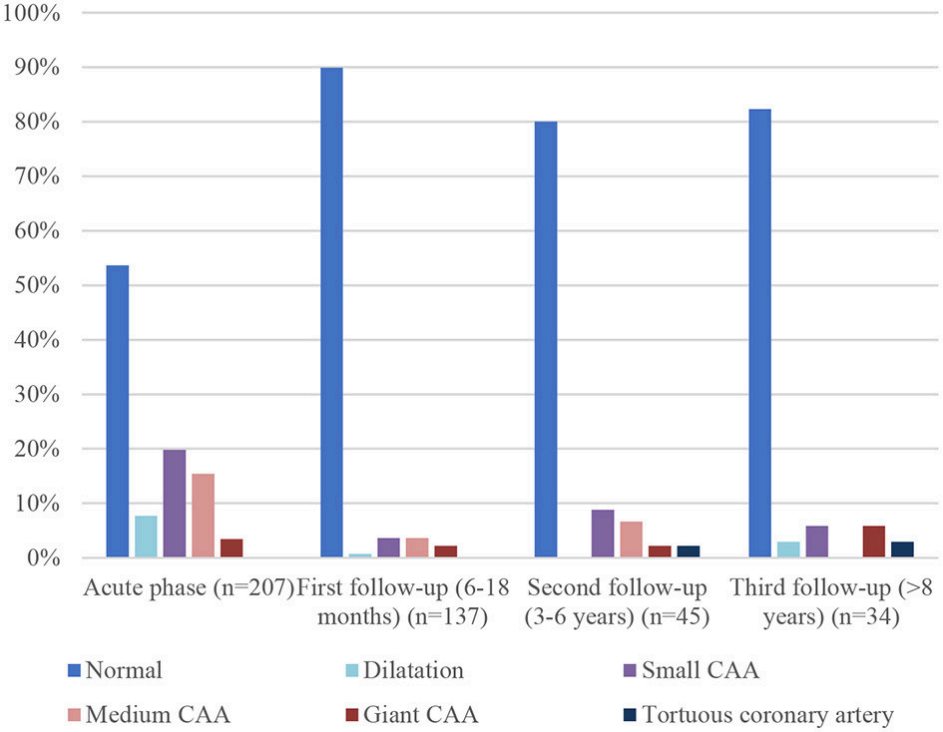
Comparison between initial coronary artery diameter and at different time.

Comparison between initial coronary artery diameter and at different time frames is shown in Figure 4. 89.8% of patients (*n* = 123) had normal coronary artery at first follow-up (6–18 months) echocardiography, compared to 82.3% (*n* = 28) at the last one. This is not related to recurrence of cardiac abnormalities but to a longer duration of the follow-up of patients with cardiac sequelae.

The first follow-up echocardiography (6–18 months) showed normal coronary artery in almost 90% of patients, compared to 34.3% in the initial one. This large difference is related to CAA and coronary dilatation or perivascular brightness regression: of the 96 patients with initial changes in coronary arteries diameter, 84 (87.5%) had only a transient abnormality and CAA or dilatation regressed over time. Regression occurred mostly within 3 months (range 2 weeks to 19 years) and only 7 of the 84 patients (8.3%) with transient abnormality had regression 2 years or more after the acute phase. Dilatations were transient in 93.7% of cases (15 patients of the 16 with initial dilatation), small CAA in 92.6% (38 of the initial 41), medium CAA in 84.3% (27 of the initial 32). Of the seven patients with giant CAA, two showed complete resolution, three partial regression, and the last two stayed giant.

Absence of IVIG in the acute phase was associated with less regression rate (57.1 vs. 92.2%, *p* = 0.07), and this association was especially important for giant CAA persisting at last echocardiography (0.5% of giant CAA in IVIG group vs. 12.5% if no IVIG was given during the acute phase, see Table 3). In addition, boys had greater z-score at last follow-up, statistically significant for the LAD (boys: median z-score 1.28, range 0.06–18.4/girls: median z-score 0.9, range 0.01–2/*p* = 0.03).

Complications of KD during follow-up occurred in 8 patients (3.8%). The most frequent complication was recurrence of KD (*n* = 5, 2.4%). Of those with recurrence, the 2nd onset of KD happened within 1 year of the acute phase in 3 cases (60%) and within 1 to 5 years for the 2 (40%). MI occurred in 3 patients (1.4%): one patient had antero-septal MI within 1 year of the onset of KD, the other two had signs of ischemia on stress-MRI or myocardial perfusion scintigraphy 5–10 years after KD. Surgery was performed in one of them (coronary artery reimplantation). MI affected only children with initial CAA. Twenty-nine patients were treated with ASA for 1 year or more (14%) and 7 for more than 10 years (3.4%). We found no statistically significant predictive factor for the occurrence of long term complication. However, the absence of IVIG treatment during the acute phase was associated with more complications during the follow-up, but with no statistically significant difference (No IVIG: 12.5% of complications vs. IVIG: 3.6%, *p* = 0.09).

## Discussion

This is the 1st study of medium to long-term follow-up of children diagnosed with KD at our Institution. The epidemiological characteristics, with a majority of patients <5 years of age with predominance of male gender and the seasonal trends, were similar to those reported in other studies from other countries (9, 14, 33–35). Incomplete KD rates were higher than expected, with 29.5% of incomplete cases compared to 20% in other studies. This difference may be associated with a high index of suspicion of this presentation of the disease at our Institution. However, patients with incomplete KD were identified and treated later than the other, highlighting the difficulty in identifying these patients. In any case, it is crucial for physicians to maintain a high index of suspicion for every child presenting with symptoms suggestive of KD. Increase in incomplete KD over time was also reported in other articles (5, 10).

The occurrence of KD has increased over time, as found in other studies (4, 5). Part of this increase may be related to a better diagnosis of KD associated with a highest awareness of the disease and an improvement in the imaging modalities.

Many non-specific clinical features were present in addition to the diagnostic criteria. This polymorphous presentation of KD makes the diagnosis even more challenging, with a large differential diagnosis.

The majority of children received treatment before the 7th day of illness, but there were still 22.7% of patients with a late therapy due to late diagnosis. This rate in delayed treatment is similar to other studies, but it is crucial to improve diagnosis and early therapy as we found that IVIG treatment during the acute phase is associated with greater regression rate and, thus, with less cardiac sequelae.

Other therapies were used in the acute phase, such as corticosteroids, IL-1 receptor antagonist or TNF inhibitor. The role of those treatments is not adequately assessed yet and it would be useful to clarify their indications.

The proportion of IVIG non-responders was comparable to other studies (32). We found no differences in terms of gender or age between IVIG responders and non-responders, as seen in other studies (29, 36).

Treatment of patients who fail to respond to initial therapy is not fully standardized but American Heart Association recommend retreatment with à 2nd infusion of IVIG (evidence level B). In our study, the majority of patients received a 2nd dose of IVIG but further studies would be needed to define clearly the best therapy for the non-responders.

The proportion of patients with abnormal echocardiography at diagnosis was high (65.7%). This number is high because it includes perivascular brightness. If we look only at coronary artery diameters, 46.4% patients developed abnormalities, with 7.7% of dilatation and 38.6% of CAA. This number of dilatation and CAA is higher than expected (8, 18, 33) and is a consequence of an increased recognition of these lesions, as we used z-score in this study. Indeed, it has been established that z-score allow for better evaluation of the severity of coronary artery changes by correcting for BSA, unlike the Japanese criteria that underestimate coronary involvement (1, 32, 37, 38). Z-score calculations changed the degree of coronary artery involvement from the initial echocardiographic report in more than 50% of patients, which had underestimated the coronary artery lesion in the majority of cases. This fact shows how essential the z-score calculations is in KD. However, z-score use has to be done with caution, as it is proved that small error in measurement of the coronary artery diameter can produce large difference in z-score calculation (32). Furthermore, accurate measurement of BSA can be difficult in irritable young children. As seen in other studies, patients with atypical ages for KD (<1 or >10 year of age) had a higher incidence of coronary artery dilatations and aneurysms (10, 34). Sites of coronary artery lesions were comparable to other study: the left coronary artery (LAD > LMCA) was more affected than the RAD (39).

Pericarditis rate was in agreement with other studies (18). As expected, pericardial involvement was transient and no chronic pericarditis occurred.

Valvular involvement was found in almost 20% of patients in this study. Valvular disease rate varies widely in literature (from 1 to 25%) (2, 32). This large difference is probably related to variable definitions of valvular involvement. In our study, we considered even slight abnormalities without clinical consequences as valvular disease. As expected, mitral valve was affected in the vast majority of cases (32).

The majority of our patients with coronary diameter changes had good outcome and more than 85% of CAA and dilatations regressed after the acute phase. This regression rate was higher than expected, with 50–67% reported in literature (1). This difference is probably due to an increased recognition of coronary artery changes, associated with the use of z-scores in spite of diameters with no correction for BSA. As seen in other studies, CAA resolution occurred mostly within 2 years after onset of KD.

Absence of IVIG in the acute phase was associated with less regression rate, which highlight IVIG importance in KD treatment. Additionally, male gender was associated with greater z-score on LAD. Thus, close follow-up of male and patients who did not received IVIG is crucial.

Complications after the acute phase documented in our patient charts were rare (3.8%) and only 3 patients suffered from MI during the study. Recurrence of KD was also rare, as seen in other countries (21). Furthermore, mortality after the acute-phase of KD was not higher than in the general population, and the increase in mortality rate among males with cardiac sequelae reported in a study was not confirmed here (26). However, it would be useful to continue follow-up of patients with a history of KD to confirm that their long-term prognosis remains good. Indeed, at the end of the study period, the oldest patient was only 36 years of age. It is therefore difficult to define exactly the cardiovascular prognosis of patients with a history of KD when they reach middle age. Further trials would be crucial to determine whether patients with prior KD have higher cardiovascular risk and could benefit from aggressive prevention of known and modifiable risk factor for MI (diabetes mellitus, hypertension, dyslipidemia, smoking, obesity).

The present study must be viewed in the light of some limitations. First, it has the limitations of a retrospective study, with lacking or incomplete information in the patient's medical record. Second, the guidelines for KD, and therefore the management of patients at our Institution, changed throughout the study period, which makes the follow-up of the patients included in this article not totally standardized. And as the study time frame is long, the follow-up of some children is incomplete, prematurely interrupted due to patients moving or transferred to adult cardiologist at a different center as the patients grow-up. Third, there is a bias of selection during the follow-up at the different times frames, as the follow-up of patients with transient CAA was shorter than the one with cardiac sequelae.

Finally, it is a single-center study and extrapolation of our results needs cautious interpretation. However, as it describes all patients at a single institution during a long-time frame, it offers a global picture of KD and its evolution in our region.

## Conclusion

In conclusion, this study shows that medium to long-term prognosis after usual treatment of KD is excellent and the majority of children do not have any cardiac sequelae or suffer from complication during the follow-up. Risk factors for poorer outcome are male gender, atypical age and absence of IVIG infusion during the acute phase. It is therefore crucial for physicians to have a high index of suspicion among children having those characteristics and offer them a strict and standardized follow-up.

Despite numerous published studies, there remains a lot unknown about the clinical features and medium to long terms outcomes in children with KD. Thus, caution is necessary during follow-up and minimization of known cardiovascular risk factor is recommended for every patient with a history of KD. Further prospective multicenter studies are warranted in order to elucidate all residual interrogations about this disease and to clarify long-term outcome of KD survivors.

## Data Availability Statement

The raw data supporting the conclusions of this manuscript will be made available by the authors, without undue reservation, to any qualified researcher.

## Ethics Statement

This study was approved by the institutional ethics committee (Commission cantonale d'éthique de la recherche sur l'être humain). Informed consent was waived by ethics committee as this is a retrospective study based only on medical files, that covers a period longer than 30 years.

## Author Contributions

MdL performed the study and wrote the paper. SdB did statistical analysis and reviewed the paper. MH contributed to the design of study and reviewed the paper. NS designed the study with MdL supervised the study and reviewed the paper.

### Conflict of Interest Statement

The authors declare that the research was conducted in the absence of any commercial or financial relationships that could be construed as a potential conflict of interest.

## Abbreviations

ASA: Aspirin
BSA: Body Surface Area
CAA: Coronary Artery Aneurysm
IVIG: Intravenous Immunoglobulin
KD: Kawasaki Disease
LAD: Left Anterior Descending Coronary Artery
LMCA: Left Main Coronary Artery
MI: Myocardial Infarction.
